# Plants in the Light of Ionizing Radiation: What Have We Learned From Chernobyl, Fukushima, and Other “Hot” Places?

**DOI:** 10.3389/fpls.2020.00552

**Published:** 2020-05-08

**Authors:** Timothy A. Mousseau, Anders Pape Møller

**Affiliations:** ^1^Department of Biological Sciences, University of South Carolina, Columbia, SC, United States; ^2^SURA/LASSO/NASA, ISS Utilization and Life Sciences Division, Kennedy Space Center, Cape Canaveral, FL, United States; ^3^Ministry of Education Key Laboratory for Biodiversity Science and Ecological Engineering, College of Life Sciences, Beijing Normal University, Beijing, China; ^4^Ecologie Systématique Evolution, Université Paris-Sud, CNRS, AgroParisTech, Université Paris-Saclay, Orsay, France

**Keywords:** plants, ionizing radiation, Chernobyl, Fukushima, mutation

## Abstract

Perhaps the main factor determining success of space travel will be the ability to control effects of ionizing radiation for humans, but also for other living organisms. Manned space travel will require the cultivation of food plants under conditions of prolonged exposure to ionizing radiation. Although there is a significant literature concerning the effects of acute high dose rate exposures on plant genetics, growth, and development, much less is known concerning the effects of chronic low dose irradiation especially those related to the impacts of the high energy protons and heavy ions that are encountered in the space environment. Here, we make the argument that *in situ* studies of the effects of radionuclides at nuclear accident sites (e.g., Chernobyl and Fukushima), atomic bomb test sites, and areas of naturally high radiation levels, could provide insights concerning the mechanisms of radiation effects on living systems that cannot be assessed short of conducting research in space, which is not yet feasible for large scale, long term, multigenerational experiments. In this article we review the literature concerning the effects of chronic low-dose rate radiation exposure from studies conducted in Chernobyl, Fukushima, and other regions of the world with high ambient radiation levels (parts of India in particular). In general, mutation rates and other measures of genetic damage are considerably elevated, pollen and seed viability are reduced, growth rates are slower, and the frequency of developmental abnormalities is increased, although there is considerable variation among taxa for these effects. In addition, there are interactions between radiation and other environmental stressors (e.g., temperature, drought, heavy metals) that may play important roles in determining susceptibility to radiation induced stress.

## Introduction

On this planet, ionizing radiation stems primarily from geological processes and is the result of the decay of radionuclides in the ground. For humans, the largest doses come from the inhalation of radon gas, a decay product of radium, which in turn is derived from the decay of uranium and thorium, which are relatively abundant in the earth’s crust. As an aside, natural radon emissions are thought to be a leading cause of lung cancer (Lubin and Boice, 1997) reinforcing the relationship between radiation and cancers. Another important radionuclide is potassium-40 which has a very long half-life of over a billion years. Uranium and thorium decay products are generally alpha and beta particle emitters making them relatively difficult to detect and measure while ^40^K is both a beta and gamma radiation emitter and can thus be easily measured using simple instruments (e.g., a Geiger counter or gamma spectrometer).

Most studies of radiation effects on plants have been conducted under laboratory or highly controlled conditions (Caplin and Willey, 2018). Most often seeds or growing plants are treated with an external gamma source, often cobalt-60, then compared to controls (e.g., Marcu et al., 2013). Findings among studies vary considerably, from stimulation of growth at low doses to negative effects at higher doses. In most cases, radiation treatment levels are extremely high (often 50–300 Gy, e.g., Maity et al., 2005), far beyond any natural conditions normally found in space or on this planet, apart from perhaps the extremely rare gamma-ray bursts related to supernova implosions (Kumar and Zhang, 2015), which are hypothesized to have led to several extinction events over the eons (Thorsett, 1995). In addition, because most studies employ an acute, high dose rate exposure to gamma rays alone, these studies may have limited relevance for issues related to the effects of radionuclides in the soil from natural or anthropogenic sources (e.g., nuclear accidents or atomic bomb testing fallout). Chronic, low dose rate with multigenerational exposure is the norm. Chronic doses associated with the space radiation environment may differ even more from exposure to gamma rays.

In space, and on Mars, the radiation environment consists of the solar electromagnetic spectrum and a diverse array of charged particles from both within and outside our solar system (Nelson, 2016). Terrestrial and even low earth orbit environments are largely shielded from these sources because of the Earth’s magnetosphere and atmosphere which deflect most of the heavy ions stemming from galactic cosmic radiation. Because of this, it is extremely difficult to conduct experiments on Earth that mimic the space environment for biologically relevant time periods.

One approach that has been used extensively has been to expose biological materials to artificially generated heavy ions. The main artificial source for heavy ions on earth come from linear accelerators like the Relativistic Heavy Ion Collider (RHIC) at DOE’s Brookhaven National Laboratory (Alessi et al., 2010) where NASA has established a space radiation laboratory (NSRL) to specifically generate the sorts of heavy ions found in cosmic radiation (La Tessa et al., 2016). Although this facility is invaluable, it is limited to generating very short duration exposures of minutes to hours. Thus, most experiments must trade off low dose rate for an acute high dose. Such a trade-off most certainly leads to a lack of realism making extrapolation to prolonged space flight conditions difficult if not impossible. That is not to say such experiments are without merit: clearly this is not the case. But it must be recognized that both total dose and dose rate, as well as developmental stage of exposure, are known to affect biological responses (e.g., Russell et al., 1958; Vilenchik and Knudson, 2000) and must be considered in any experimental design.

Here, we are advocating an alternative, more realistic approach, that uses a mixture of radionuclides *in situ* to simulate chronic exposure to both charged particle and photon radiation sources. We suggest that prolonged exposure to alpha and beta particles, and gamma photons, is a more realistic proxy for experimental studies of space radiation than acute high doses of heavy ions or gamma rays alone in an artificial, laboratory setting. Although far less energetic than the heavy ions found in Galactic Cosmic Radiation (GCR), alpha and beta particles do have mass and their interactions with biological molecules more closely resembles that expected for GCR than gamma photons alone. Certainly, if ingested, alpha and beta generating radionuclides can deliver substantial doses to intracellular structures (e.g., DNA, lipids), and there are many common radionuclides capable of delivering substantial gamma dose rates both externally and internally (e.g., ^137^Cs and ^60^Co). Most importantly, experimental treatments can be calibrated to deliver a variety of dose rates over multiple generations thus permitting a simulation of the conditions experienced during space flight and life on Mars.

That said, there are many challenges to using radionuclides for environmental experiments. The greatest challenge comes from their toxicity and the difficulty of clean-up after use. Half-lives of commonly available radioisotopes (e.g., ^137^Cs, ^90^Sr, ^238^U) are often quite long (30, 29, and 4.5B years, respectively) thus requiring expensive remediation of experimental plots after use even if one could get permission to conduct such an experiment to begin with. But this is far less a challenge than simulating GCR for prolonged experimental durations.

Here, we are advocating an alternative approach that offers many opportunities and few of the costs or drawbacks suggested above. Nuclear accident sites (e.g., Chernobyl or Fukushima), atomic bomb test sites (e.g., the Marshal Islands or Semipalatinsk), and naturally occurring radioactive landscapes (e.g., Kerala, India, Ramsar, Iran) potentially provide a setting for conducting large scale experiments without the need for major remediation following the experiments. Specific locations can be selected for a particular ambient radiation level that includes a mix of alpha, beta, and gamma sources, and dose rates can be selected or manipulated to include very low to relatively high doses (e.g., in excess of 1 mGy/h in Chernobyl) to assess dose-response relationships which is an important aspect of testing for causality in such studies (Shapiro, 2008). In some places, it is possible to manipulate the radiation environment by transporting contaminated soil to experimental plots (e.g., Chernobyl) or diluting substrates to achieve a desired dose rate. Given that the Chernobyl Exclusion Zone is highly heterogeneous with respect to contamination levels, it is easily possible to identify areas where both treatment and “control” plots could be located with a few hundred meters of each other. We have utilized this feature of the Chernobyl zone to compare highly radioactive to relatively “clean” areas at multiple locations thus permitting multiple paired-comparisons across the region (e.g., studies of decomposition, tree growth, abundance, and diversity; Mousseau et al., 2013, 2014, among many others). Field plots and/or greenhouses with highly contrasting radiation conditions are certainly feasible and have been used on occasion in the past (e.g., Dmitriev et al., 2011). Our perspective on this topic is not original and has been suggested before (e.g., Caplin and Willey, 2018) but it bears repeating given the near total lack of investment in this research area.

Similar to accident sites, atomic bomb test sites offer a wide range of radionuclides and radiation levels that could be exploited for experimental tests of radiation effects on plant growth. For example the United States detonated 67 atomic bombs at the Marshall Islands and the Soviet Union tested 456 bombs in Semipalatinsk region of Kazakhstan. Vast quantities of radionuclides persist in these regions yet to our knowledge, there have been very few studies concerning the biological impacts of the fallout. Studies of organisms living in these regions could offer insights concerning evolved adaptive responses as most testing ended more than 60 years ago in these areas, or about twice as long since the Chernobyl accident which occurred in 1986.

In contrast to atomic bomb test sites and accidents at nuclear facilities, there are geographic regions where naturally occurring radiation can reach very high levels (three orders of magnitude above global mean levels), as found in India, China, Iran, Turkey, Namibia, and Brazil among others (Møller and Mousseau, 2013b). In addition, there are equally many sites with high levels of radiation in the oceans with thermal vents and their associated unique biodiversity being a well-known example (e.g., Cherry et al., 1992; Jolivet et al., 2004). Maximum terrestrial levels of radioactivity reach as high as 29.7 μSv/h in Ramsar, Iran, 22 μSv/h in Morro do Ferro, Minas Gerais, Brazil, 12 μSv/h in Mombasa, Kenya, 10 μSv/h in Lodeve, France, 4.0 μSv/h in Kerala, India, 4.0 μSv/h in Tamil Nadu, India, and 0.7 μSv/h in Yangjiang, China (Ghiassi-Nejad et al., 2002). These levels of radiation are 20-fold less than the maximum levels today at Chernobyl but they have existed for geological time periods thus providing ample opportunity for evolved adaptations to ionizing radiation.

For more than two decades we have conducted research on the biological effects of radiation stemming from radionuclides in Chernobyl, and in Fukushima since 2011. We have also surveyed the literature and conducted meta-analyses of the findings from research conducted in naturally radioactive regions of the planet including parts of India, Iran, Brazil, and elsewhere. Studies in all these environments have provided a plethora of novel insights concerning the impact of ionizing radiation on natural systems that is at once much more realistic of both terrestrial and space environments than traditional laboratory studies, given the chronic exposure to a mix of radiation types. In addition, because many of the findings reported here reflect long term responses to ionizing radiation, this permits testing for adaptive responses of potential utility for the development of plant resources for space flight.

Here we review the impact of terrestrial ambient radiation on plants. First, we review mutation rates in plants in Chernobyl and other naturally radioactive places around the world (Møller and Mousseau, 2013b, 2015). Second, we review impacts of ionizing radiation on plant growth and morphological abnormalities. Third, we review the impact of ionizing radiation on plant reproduction. Fourth, we review the literature on how other organisms through herbivory and parasitism impact flowering plants and their performance. Fifth, we review studies of the effect of ionizing radiation on plant ecosystems (Santos et al., 2019). Finally, we provide future directions for research on the impact of ionizing radiation on flowering plants.

## Ionizing Radiation and Mutation Rates in Plants

Elevated mutation rates are a key feature of ionizing radiation (Muller, 1950). Previous studies have shown increased mutation rates in some, but not in all plants (Kovalchuk et al., 2000; Møller and Mousseau, 2013b; Aguileta et al., 2016). In an extensive summary of radiation effects stemming from natural sources all around the planet, Møller and Mousseau (2013b) demonstrated that plants, as a group, had effects of ionizing radiation that were almost an order of magnitude higher than in animals (see Table 2, Møller and Mousseau, 2013b). Mean effect size for effects of radiation on mutations and other traits weighted by sample size was 0.749 (95% CI 0.570–0.878), while in animals this was only 0.093 (95% CI 0.039–0.171) (Møller and Mousseau, 2013b, p. 246). Effect size in such metanalyses is a scaled measure of the correlation between the dependent and independent variables. These strong effects may reflect the sedentary life history of most plants or differences in the ability to repair the damage inflicted by ionizing radiation. The fact that exposure to radiation is continuous over many generations may also play a significant role as the effects may be compounded by mutation accumulation across multiple generations as has been suggested in studies of other groups (e.g., Garnier-Laplace et al., 2015; Omar-Nazir et al., 2018). It is notable that many natural sources have dose rates that are relatively low, with the highest on the order of 200 mGy/y (most are substantially lower), suggesting that even very low dose rates can have significant repercussions for organismal functioning.

In a subsequent metanalysis, Møller and Mousseau (2015) performed a comprehensive study of all known experimental studies of radiation effects for organisms living under the influence of radio-contaminants stemming from the Chernobyl disaster. As with the analysis of natural radiation sources (Møller and Mousseau, 2013b), this analysis found that plants showed significantly higher effect sizes than animals [0.749 (95% CI 0.570–0.878) vs 0.089 (95% CI 0.071–0.108); *Q*_*b*_ = 5.044, df = 1, *p* = 0.025] although the differences were not nearly as dramatic as those seen in areas of naturally high radiation. This difference between studies could reflect the much higher sample sizes for the latter study or perhaps the impacts of evolved adaptations to radionuclides although in general there is very little evidence to suggest that higher organisms (including plants) show much in the way of genetically based adaptive responses to ionizing radiation in the Chernobyl region (Møller and Mousseau, 2016).

A detailed listing of the plant studies included in Møller and Mousseau (2015) is given in Table 1. Effect sizes varied considerably among species and interspecific differences could be explained by differences in resistance to radiation due to physiological mechanisms, DNA repair ability and other factors such as life history or mode of reproduction. Differences between plants and animals could partly be due to the sedentary nature of plants, which unlike animals are unable to temporarily or permanently move away from the most contaminated areas, or differences in genome size and ploidy. Much further work is needed to address these questions.

**TABLE 1:** Mutation and genetic damage rates in Chernobyl plants.

Species	Variable	Effect size −Zr	Range of radiation (mGy)	Reference
*Achillea millefolium*	Abnormal divisions	−0.41	0.01–1.12	Kordium and Sidorenko, 1997, p. 42, 44
*Arabidopsis thaliana*	Mutant plants	1.61	2.40–57.6	Abramov et al., 1992, p. 22
*Avena sativa*	Aberrant cells	0.55	5.32–47.8	Geraskin et al., 2003, p. 163
*Calamagrostis epigejos*	Abnormal spores	1.50	0.01–1.12	Kordium and Sidorenko, 1997, p. 44
*Calamagrostis epigejos*	Abnormal pollen	0.72	0.01–1.12	Kordium and Sidorenko, 1997, p. 42
*Chamaenerium angustifolium*	Abnormal divisions	1.20	0.01–1.12	Kordium and Sidorenko, 1997, p. 44
*Chamaenerium angustifolium*	Abnormal spores	1.09	0.01–1.12	Kordium and Sidorenko, 1997, p. 44
*Chamaenerium angustifolium*	Abnormal pollen	1.48	0.01–1.12	Kordium and Sidorenko, 1997, p. 44
*Crepis tectorum*	Chromosome aberrations	0.03	0.01–19.2	Shevchenko et al., 1995, p. 698
*Crepis tectorum*	Karyotype changes	−0.25	0.01–19.2	Shevchenko et al., 1995, p. 698
*Elytrigea repens*	Abnormal divisions	−0.22	0.01–1.12	Kordium and Sidorenko, 1997, p. 44
*Elytrigea repens*	Abnormal spores	0.07	0.01–1.12	Kordium and Sidorenko, 1997, p. 42
*Elytrigea repens*	Abnormal pollen	−0.45	0.01–1.12	Kordium and Sidorenko, 1997, p. 42
*Hordeum vulgare*	Aberrant cells	1.15	5.32–47.8	Geraskin et al., 2003, p. 163
*Hypericum perforatum*	Abnormal pollen	2.41	0.01–1.12	Kordium and Sidorenko, 1997, p. 44
*Jasione montana*	Abnormal divisions	1.42	0.01–1.12	Kordium and Sidorenko, 1997, p. 44
*Jasione montana*	Abnormal spores	1.35	0.01–1.12	Kordium and Sidorenko, 1997, p. 44
*Jasione montana*	Abnormal pollen	0.98	0.01–1.12	Kordium and Sidorenko, 1997, p. 44
*Oenothera biennis*	Abnormal pollen	3.80	0.01–1.12	Kordium and Sidorenko, 1997, p. 44
*Phragmites australis*	Chromosome fragments	0.99	0.01–9.30	Gudkov et al., 2006, p. 7
*Phragmites australis*	Chromosome bridges	1.05	0.01–9.30	Gudkov et al., 2006, p. 7
*Pinus sylvestris*	Segregation distortion	0.66		Shevchenko et al., 1996, p. 124
*Pinus sylvestris*	Point mutations	0.78		Shevchenko et al., 1996, p. 124
*Pinus sylvestris*	Null mutations	1.01		Shevchenko et al., 1996, p. 124
*Pinus sylvestris*	Duplications	1.05		Shevchenko et al., 1996, p. 124
*Pinus sylvestris*	Cells with chromosomal aberrations	1.63		Shevchenko et al., 1996, p. 124
*Pinus sylvestris*	Chromosome aberrations	0.85–1.35	0.11–385	Kal’chenko and Fedotov, 2001
*Pinus sylvestris*	Changes per locus	1.02	0.11–8.10	Kal’chenko and Fedotov, 2001, p. 346
*Pinus sylvestris*	Segregation distortion	1.41	0.11–8.10	Kal’chenko and Fedotov, 2001, p. 347
*Pinus sylvestris*	Excess S over F	1.14	0.11–8.10	Kal’chenko and Fedotov, 2001, p. 348
*Pinus sylvestris*	Mutation frequency in endosperm	1.42	0.11–8.10	Kal’chenko and Fedotov, 2001, p. 346
*Pinus sylvestris*	Seed yield	−0.74	3.9–385	Kalchenko and Rubanovich, 1993, p. 1206
*Pinus sylvestris*	Allozyme mutations	1.14	3.9–385	Kalchenko and Rubanovich, 1993, p. 1208
*Pinus sylvestris*	Endosperm mutations	1.57	3.9–385	Kalchenko and Rubanovich, 1993, p. 1209
*Pinus sylvestris*	Seedling mutations	1.53	3.9–385	Kalchenko and Rubanovich, 1993, p. 1209
*Pinus sylvestris*	AFLP mutations	0.42	15.6–29.0	Kuchma et al., 2011, p. 25
*Plantago major*	Abnormal divisions	0.82	0.01–1.12	Kordium and Sidorenko, 1997, p. 44
*Plantago major*	Abnormal spores	−0.62	0.01–1.12	Kordium and Sidorenko, 1997, p. 42
*Plantago major*	Abnormal pollen	0.38	0.01–1.12	Kordium and Sidorenko, 1997, p. 42
*Secale cereale*	Aberrant cells	0.71	5.32–47.8	Geraskin et al., 2003, p. 164
*Secale cereale*	Severity of damage	0.38	5.32–47.8	Geraskin et al., 2003, p. 160
*Secale cereale*	Multiple damage	0.38	5.32–47.8	Geraskin et al., 2003, p. 160
*Secale cereale*	Cytogenetical damage of root meristem	1.64	7.10–65.6	Ziablitskaia et al., 1996, p. 502
*Secale cereale*	Cell aberrations	0.83	7.10–65.6	Ziablitskaia et al., 1996, p. 502
*Trifolium arvense*	Abnormal divisions	1.65	0.01–1.12	Kordium and Sidorenko, 1997, p. 44
*Trifolium arvense*	Abnormal spores	1.38	0.01–1.12	Kordium and Sidorenko, 1997, p. 42
*Trifolium arvense*	Abnormal pollen	2.99	0.01–1.12	Kordium and Sidorenko, 1997, p. 42
*Triticum sativum*	Aberrant cells	0.78	5.32–47.8	Geraskin et al., 2003, p. 164
*Triticum sativum*	Microsatellite mutations	0.19	0.001–1.5	Kovalchuk et al., 2000, p. 583
*Typha angustifolia*	Allele number	0.93	0.13–7.52	Tsyusko et al., 2006, p. 2620
*Typha angustifolia*	Allele number	0.30	0.13–7.52	Tsyusko et al., 2006, p. 2620
*Typha latifolia*	Allele number	0.51	0.13–7.52	Tsyusko et al., 2006, p. 2620

*Effect size refers to the size of the radiation effect with positive numbers denoting higher effects with increasing ambient radiation levels. Table modified from Møller and Mousseau (2015). All effects reported above were significantly different from zero based on calculations of 95% confidence intervals reported in figure 2 and supplemental information of Møller and Mousseau (2015).*

De Micco et al. (2011), Caplin and Willey (2018), and Arena et al. (2014) have conducted comprehensive reviews of experimental studies related to radiation sources of many types on plant mutations, including several studies conducted during space missions. Other experimental radiation sources included gamma rays, heavy ions of all sorts, and neutrons. Overall, the findings reinforce the expectation that heavy ions are a significant mutagen for plants and are very likely to pose significant challenges for plant cultivation during long term space travel.

## Impacts of Ionizing Radiation on Morphological Abnormalities

Plants and animals living in elevated radiation environments have been shown to have higher incidences of abnormalities including pheno-deviants and degrees of fluctuating asymmetry. Møller (2002) showed for 15 out of 15 studies of plants and animals studied in Chernobyl and in less contaminated control areas that the level of developmental instability in all cases was higher in Chernobyl than in control areas. That was also the case for three species of plants with the degree of abnormality in petal length and number deviating from the situation in uncontaminated controls (Møller, 1998). Zakharov and Krysanov (1996) listed abnormalities in leaves of a number of different plant species including differences in length of right and left morphological characters, but also differences in the number of leaflets on leaves of the right and left side of the symmetry axis, and differences in the variance in right and left characters of plants. The species studied with respect to leaf asymmetry were soy beans *Glycine max*, flax *Linum usitatissimum*, and robinia *Robinia pseudacacia*. While these levels of abnormalities were small, Møller and Mousseau (2003) showed that such abnormalities could be associated with reduced viability and reproductive success in other organisms.

## Plant Growth and Radiation

There have been several studies of tree growth and morphological aberrations in the Chernobyl region. For example, Mousseau et al. (2013) demonstrated significant decreases in growth rates of Scots pines (*Pinus sylvestris*) living in contaminated areas using a dendrochronological analysis of 105 trees across the spectrum of radiation levels in the Chernobyl Exclusion zone. Trees were selected to be at least 35 years old at the time of the study (2009) so that there was a record of growth from at least 10 years prior to the accident. Growth rates from both before and after the accident in 1986 were estimated using growth rings which are very easily measured for pine trees. A longitudinal analysis demonstrated very large decreases in growth rates in the most radioactive areas for 3 years following the accident, followed by smaller decreases particularly in years of drought. This was likely a consequence of the very high doses experienced by the trees at the time of the accident which declined dramatically in subsequent years. Scots pines have been shown to be particularly vulnerable to the effects of radiation and recruitment to areas above 50 μGy/h ambient radiation has been minimal even to this day. Several studies have demonstrated that younger trees were particularly vulnerable to the effects of radiation (e.g., Tulik and Rusin, 2005; Mousseau et al., 2013) with significant changes in growth form and wood quality observed.

Many of the pines in the most contaminated areas of Chernobyl show dramatic changes in the morphology with peculiar branching reflecting damage to the meristems at the time of the accident (e.g., Kozubov and Taskaev, 2002). Not coincidentally, Japanese red pine (*Pinus densiflora*) and Japanese fir (*Abies firma*) trees both showed developmental abnormalities similar to those seen in Chernobyl following the Fukushima accident (Watanabe et al., 2015; Yoschenko et al., 2016). This convergence in effects provides strong support for the hypothesis that exposure to radiation during development was the causal factor underlying these developmental aberrations. At present, little is known concerning the genetic or physiological mechanisms associated with these effects.

It is not known the degree to which the effects seen in Chernobyl are the result of direct effects on the trees themselves versus indirect effects mediated via other biotic factors. For example, studies of decomposition of plant material and soil invertebrate activity also show significant declines in areas of high contamination levels (Mousseau et al., 2014; Bezrukov et al., 2015), and this in turn could result in lower nutrient recycling rates with consequent effects on plant growth. It seems likely that both biotic and abiotic stressors interact with radiation to affect plant growth in these “natural” systems.

Overall there have been very few studies of the effects of radiation on plants related to the Fukushima event. Notable exceptions include studies of tree growth mentioned above, and studies of rice by Hayashi et al. (2014, 2015) and Rakwal et al. (2018) that have suggested effects on plant growth, DNA repair, stress responses, and an array of gene expression responses. These studies are likely to be very useful for further investigations of mechanisms associated with genetic and physiological responses to chronic radiation exposures.

Desiderio et al. (2019) conducted laboratory studies of X-ray and gamma ray exposure on tomato “hairy root” cultures and found significant effects on stress response activation and protein metabolism when cells were exposed to high radiation levels (>5 Gy) but not at lower exposures (0.5 Gy). Arena et al. (2019) suggested that *Solanum lycopersicum* seeds were not negatively affected by exposure to high doses from Ca ions because seedlings that germinated following exposure had better photochemical efficiency than controls and produced larger fruits. However, this seemingly positive effect may have resulted from the tradeoffs associated with producing fewer fruits. Biermans et al. (2015) tested for the effects of alpha particle exposure on *Arabidopsis thaliana* from ^241^Am and found negative impacts on photosynthesis performance and carbon assimilation that likely resulted from redox balance declines.

## Ionizing Radiation and Plant Reproduction

Ionizing radiation may impact reproductive organs and gametes and hence reduce plant reproduction. This may delay phenology and hence the timing of reproduction. The difference between the number of buds and the number of flowers may also be affected by radiation with a relatively larger number of flowers relative to buds reflecting a relatively later timing of reproduction. Radiation may also reduce growth of plants and hence exposure of flowers to pollinators. The number and the size of flowers may affect reproductive output including seed set. We have recorded these components of reproduction in 73 species of flowering plants in Chernobyl and Fukushima in order to test whether ionizing radiation affects growth of plants.

Ionizing radiation may also affect pollen viability and hence reproductive output. Møller et al. (2016) showed for 109,000 pollen grains from 675 pollen samples from 111 species of plants at Chernobyl that there was an overall negative, but weak relationship between pollen viability and radiation. Only ploidy level and the number of nucleate cells influenced the strength of the relationship between the level of radiation and viability. Møller and Mousseau (2017) showed that germination rate of plants from Chernobyl was lower in more contaminated areas even when seeds are grown in an uncontaminated common garden. Møller and Mousseau (2017) showed no significant effects of genome size, number of chromosomes, level of ploidy or pollination by bi- or trinucleate pollen grains. Finally, seed weight decreased among sites with higher levels of ionizing radiation. In contrast, Arena et al. (2014) have suggested that there is a positive relationship between “chromosome volume” and mutation rates i.e., species with larger chromosome sizes are more vulnerable to radiation although they also suggest that polyploid species are more resistant to radiation effects because gene redundancy “protects polyploids from the deleterious effects of mutations.”

In a common garden experiment, Boratyński et al. (2016) examined germination and growth of seeds and seedling wild carrots (*Daucus carota*) collected from Chernobyl sites that varied in radiation levels by three orders of magnitude (0.08–30.2 μGy/h). The only significant predictor of germination rates was the radiation level from which the maternal plant was growing. In addition, the time to germination was longer as was the time to produce cotyledons for plants derived from more radioactive areas. Given that seeds and seedlings were grown in a common garden environment these findings suggest that exposure to ionizing radiation in the maternal generation carried over to the offspring. This could reflect genetic effects or other maternally transmitted factors in the seeds. Such transgenerational interactions are nearly ubiquitous in both plants and animals but often ignored. Such parental (and grandparental) effects must be accounted for in any experimental design (Mousseau and Fox, 1998a, b).

Experimental studies conducted in space and in the lab using heavy ions, and gamma sources, have also generally found effects on plant reproduction although the results vary depending on species and the biological endpoint studied (see Table 2, De Micco et al., 2011).

## Ionizing Radiation, Herbivory and Parasitism Impact Flowering Plants

Plants interact with herbivores, parasites, decomposers and pollinators. Hence changes in the abundance and the diversity of plant species and their growth and life history may be partially affected by these different taxa. There is considerable variation in density of herbivores across radiation gradients within the Chernobyl Exclusion Zone (Møller and Mousseau, 2013a). Roe deer *Capreolus capreolus*, red deer *Cervus elaphus*, moose *Alces alces*, and bank voles *Myodes glareolus* are abundant depending on ambient radiation. The abundance of bank voles is mainly determined by food availability and hence vegetation, but also by the interaction between food and radiation (Mappes et al., 2019). It remains to be seen if the abundance of plants depends on ambient radiation levels and the abundance of herbivores.

Fungi play important roles as decomposers and parasites. Parasitic fungi of the genus *Microbotryum* are transmitted by pollinators to flowers of caryophyllaceous plants sterilizing flowers and hence reducing reproductive output of plant hosts (Aguileta et al., 2016). Indeed, the prevalence of the anther smut increases with the abundance of butterflies, which in turn decrease in abundance with the level of ambient radiation. Interestingly, the impact of radiation on prevalence of the smut fungus depends on the abundance of pollinating butterflies as revealed by the statistical interaction. Subsequent molecular analyses revealed no dose-dependent substitution rates showing no evidence of radiation-dependent mutation rates. In fact, there was evidence of stronger purifying selection in contaminated that in non-contaminated areas.

Plant-microbe interactions play a large role in determining plant health (Berg, 2009) and success in natural settings (Reynolds et al., 2003). To date, virtually nothing is known concerning how such interactions might be influenced in a radioactive setting although field studies in Chernobyl suggest that radiation effects on decomposition may be affecting plant growth in important ways (Mousseau et al., 2014).

## Effect of Ionizing Radiation on Plant Ecosystems

The Chernobyl Exclusion Zone is characterized by predominant cover with grasses and trees following the impact of the nuclear disaster. Santos et al. (2019) used the Normalized Difference Vegetation Index (NDVI) derived from satellite images and measurements of ambient radiation from the ground. Remote sensing and Landsat satellite images across years were used to relate NDVI to background radiation. Analyses of the association between NDVI and ambient radiation measurements were made using analysis of variance and Generalized Additive Models. NDVI increased over the years after the accident in 1986 largely independent of current ambient radiation. The increase in green coverage in the exclusion zone is due to positive effects of land abandonment and reduced abundance of herbivores that surpassed the negative effects of radiation exposure on the vegetation. The Chernobyl Exclusion Zone is now dominated by grasses and shrub/trees with the latter dominated by Scots pine and silver birch *Betula pendula*. Santos et al. (2019) concluded that there were positive effects of abandonment of farmland for the abundance of some plant species, while there were negative impacts of exposure to ionizing radiation on the vegetation. Finally, Santos et al. (2019) showed that the vegetation was negatively affected by a threshold level of ionizing radiation. This suggests that level of ionizing radiation reduces the abundance of some, but not of other species of trees. While the abundance of certain tree species has increased, the abundance of flowering plants has decreased considerably.

We have surveyed plant communities in 80 sites in Chernobyl that were either uncontaminated control areas or contaminated areas varying in their degree of contamination. In general, there are strong interactions between plants and insects, with ramifications for plant fertility and recruitment, and community structure (Møller et al., 2012). This study began in 2003 and has been repeated again at the same sites in 2018. These extensive analyses of the impact of radiation on plant communities will allow for continuous assessment of plant communities in years to come. They will also allow for comparison of short- and long-term effects of ionizing radiation.

## Conclusion

Studies of genetics, development, and life history at terrestrial sites having large amounts of radionuclides offer an opportunity to experimentally investigate exposure to a wide range of ionizing radiation sources over long time periods that in some ways could be used as a proxy for studies of cosmic radiation. Current knowledge suggests that many plants are vulnerable to such exposures as evidenced by the many examples of genetic damage and developmental abnormalities not widely seen under “normal” conditions. Chernobyl, in particular, is a valuable “natural” laboratory given the relatively high radiation levels stemming from a diversity of radionuclides across its broad landscape that could be experimentally manipulated to investigate the complex ways that plants adapt and evolve to changes in a radioactive environment. In addition, paired studies at other accident sites (e.g., Fukushima), atomic bomb test sites (e.g., the Marshall Islands), and naturally radioactive regions of the world offer unique opportunities to dissect out effects of particular radionuclides and types of ionizing radiation (i.e., alpha, beta, and gamma) as well as the importance of time since start of exposure on adaptive and evolutionary responses.

## Author Contributions

TM and AM conceived and wrote the manuscript, and read and approved the final manuscript.

## Conflict of Interest

The authors declare that the research was conducted in the absence of any commercial or financial relationships that could be construed as a potential conflict of interest.
